# Use of DNA Markers for Grape Phylloxera Population and Evolutionary Genetics: From RAPDs to SSRs and Beyond

**DOI:** 10.3390/insects10100317

**Published:** 2019-09-25

**Authors:** Javier Tello, Astrid Forneck

**Affiliations:** Department of Crop Sciences, Institute of Viticulture and Pomology, University of Natural Resources and Applied Life Sciences Vienna, Konrad Lorenz Str. 24, A-3430 Tulln, Austria; javier.tello@boku.ac.at

**Keywords:** AFLP, RAPD, *Daktulosphaira vitifoliae*, genotyping, mtDNA sequencing

## Abstract

Grape phylloxera (*Daktulosphaira vitifoliae* Fitch) is a major pest of cultivated grapevines (*Vitis* spp.), occurring in virtually all viticultural regions around the world. Different grape phylloxera strains can be found at varying levels on leaves and roots on both own-rooted plants and in plants grafted onto partially resistant rootstocks. Considering its relevance for the adequate management of the pest in infested vineyards, the analysis of its genetic diversity has received considerable attention from the scientific community in the last decades. Here, we review 25 years of DNA-based molecular markers applied to the analysis of the genetic structure and the reproductive mode of grape phylloxera in its native range and in different introduced regions. The use given to RAPD, AFLP, mtDNA sequencing and microsatellite (SSR) genetic markers for the analysis of grape phylloxera diversity is discussed, and an overview of the main findings obtained after their application to different populations collected in diverse regions all around the world is shown. Lastly, we explore how recent advancements in molecular biology and in modern high throughput genotyping technologies may be applied to better understand grape phylloxera natural diversity at a molecular level.

## 1. *Daktulosphaira vitifoliae*: A Major Pest of Cultivated Grapevines

**Figure 1:**
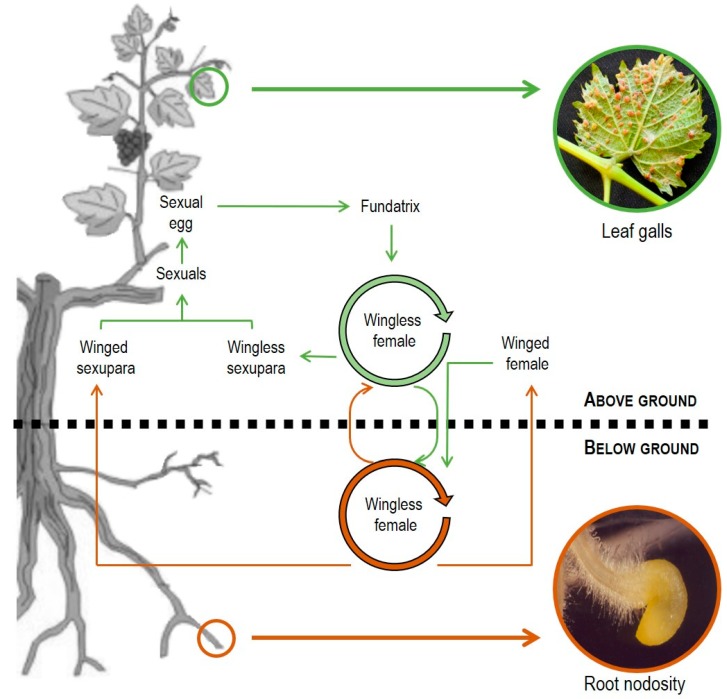
Life cycles of grape phylloxera (modified from [1]). Above- and below-ground stages are shown in green and brown, respectively.

Grape phylloxera (*Daktulosphaira vitifoliae* Fitch) is an obligate biotroph on *Vitis* spp. Its life cycle exists in many variants, but it is generally considered as holocyclic with both sexual and asexual reproductive stages (Figure 1) [1]. Briefly, and considering northern hemisphere conditions, the first generation is initiated parthenogenetically in spring when climatic and plant phenological conditions are optimal. At that time, a female *fundatrix* hatches from an overwintering fertilized egg and it moves up onto plant leaves to recommence asexual parthenogenetic reproduction. The asexually produced progeny emerges from their galls as first instars, which initiate additional galls to produce up to 3–4 asexual generations. As the season progresses, some of the wingless females move down to the roots of the vine in early spring and summer, or vice versa up to the leaves from the plant roots. Once there, they initiate root galls that induce nodosities and/or tuberosities in roots, or galls in leaves, respectively. From mid-summer to late autumn, the root-feeding wingless females produce winged individuals, which move from below ground, where they produce non-feeding offspring (male and female) that might mate. The female *oviparae* lay a single egg that will ultimately give rise to a new *fundatrix* and hence an asexual lineage. During this complex life cycle, and in a susceptible host, grape phylloxera generates serious structural damages in the plant that might eventually cause plant death after several years of infestation [2]. In roots, grape phylloxera induces the formation of organoid galls immediately behind the root tip or near the zone of elongation (Figure 1). These galls induce the generation of root nodosities (in young root tips) and/or root tuberosities (in mature roots), which serve as the insect’s nutritional basis [3,4]. In leaves, it induces the formation of histoid galls on newly expanded leaves (Figure 1) to increase the availability of nutrients and facilitate their acquisition by a complex reprogramming of vine leaf development, structure and metabolism [5]. As a result, grape phylloxera might cause severe vine decline or death by a series of mechanisms that include: (I) a removal of photosynthates that may cause loss of vine vigour; (II) root mortality by secondary pathogens entering feeding wounds (which cause additional water and nutrient stresses to the vine); and (III) indirect physiological disruption of photosynthates and water stress [2,6,7,8].

**Figure 2:**
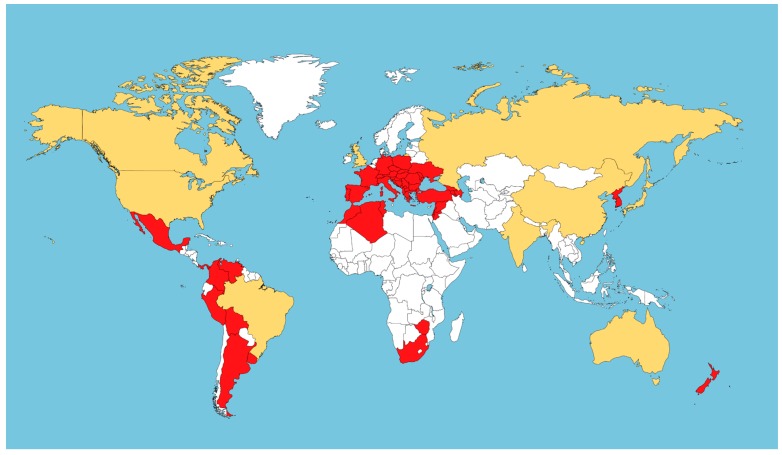
Worldwide distribution of grape phylloxera (*Daktulosphaira vitifoliae* Fitch). Countries with a national widespread distribution of the pest are shown in red; those with grape phylloxera present in specific regions in yellow. Countries with no presence of the pest or with no available data are indicated in white. The data refer to 2014 records, extracted from the Scientific Opinion on the risk to plant health posed by *Daktulosphaira vitifoliae* (Fitch), in the EU territory, with the identification and evaluation of risk reduction options (available at https://www.efsa.europa.eu/en/efsajournal/pub/3678).

Grape phylloxera is native of the eastern Rocky Mountains in North America, where it still can be found cohabiting with native *Vitis* spp. [2]. Undoubtedly, grape phylloxera was an important driving force in the evolution of American *Vitis* spp. in its natural range, acting as an antagonist on its host [9]. It has been suggested that American vines developed different adaptation mechanisms to reduce the damage potentially caused by grape phylloxera, including full resistance (no galling and no apparent effect on the vine), partial resistance (some galling happens, either on vine roots or leaves, but the extent is considerably lower compared to tolerant plants), or tolerance (the host can support a high level of leaf or root galling without apparent detrimental effect on the vine) mechanisms [9]. As a result, it has been indicated that the different processes of co-evolution between American *Vitis* spp. and grape phylloxera led to the development of two geographically diverse groups of American *Vitis* spp. with different degrees of resistance to grape phylloxera leaf and root attack: (I) a northeastern group consisting of *V. aestivalis* and *V. labrusca* (both tolerant of heavy root galling but low leaf attack), *V. riparia* (tolerant of heavy leaf galling but low root attack) and *V. cordifolia* (partially resistant to leaf and root attacks), and (II) a central and southern group consisting of *V. monticola* (heavy root galling but no leaf attack), *V. rupestris* (high leaf-galled but low root attack) and *V. berlandieri*, *V. candicans*, and *V. cinerea* (partially resistant to both type of attack) [9]. The movement of plant material between continents in the late 19th century caused the unintentional introduction of the insect into renowned wine-producing regions in Europe, where the grape phylloxera-susceptible European *V. vinifera* L. species was grown [2]. Grape phylloxera initially arrived in France [10], and from there it spread throughout Europe [2], causing a massive destruction of ca. 2.5 million ha of vineyards [11]. Genetic analyses have suggested that most of the strains causing the devastation of European vineyards initially originated from eastern North America, where *V. riparia* dominates [12]. At that moment, European viticulture was saved from extinction by the use of several *Vitis* spp. as rootstocks and for the breeding of disease-resistant inter-specific hybrids [13,14]. Nevertheless, the continuous introduction of additional *Vitis* spp. plants from North America caused the introduction of additional grape phylloxera biotypes in European vineyards [9]. As a result, intensive public and private breeding programmes were developed to generate novel hybrids from diverse accessions of North American *Vitis* spp. (predominantly *V. berlandieri* cv. Rességuier, *V. rupestris* cv. du Lot and *V. riparia* cv. Gloire de Montpellier) to be used as rootstocks [15]. In fact, most of the rootstocks that are still used in commercial grape production nowadays were generated 100 years ago as a way to combat grape phylloxera introduction [15,16]. In parallel, the inadvertent transference of grape phylloxera individuals on infested grapevine cuttings from Europe and/or North America to New World vineyards (California, South America, South Africa, Australia, Asia, etc.) led to the worldwide dispersal of the insect [17,18,19,20,21]. As a result, grape phylloxera can be found in almost all viticultural regions worldwide (Figure 2), and it is considered a major pest for the cultivated grapevine [2]. In this context, only a few relevant viticultural areas (like Chile, Cyprus, and some regions of Australia) officially remain free of grape phylloxera, mainly due to an intensive combination of surveillance, detection and quarantine programs [2].

As indicated in numerous works [22,23,24,25,26], the different level of performance and aggressiveness of diverse grape phylloxera lineages on various *Vitis* spp. hosts indicate the presence of diverse biotypes. Recently, an updated classification system of grape phylloxera biotypes has been proposed based on insect–host plant interaction mechanisms (feeding sites, preferential feeding organ and insect performance) [27]. Following this work, up to seven biotypes (A–G) can be differentiated according to their ability to establish and develop on a particular host (Table 1). As an example, biotype A strains show superior performance on nodosities and tuberosities on *V. vinifera* roots and a limited performance on nodosities on rootstock roots derived from crosses between American *Vitis* spp., whereas biotype B includes those strains with superior performance on nodosities and tuberosities on the roots of rootstocks derived from American *Vitis* spp. crossed with *V. vinifera*, but a limited performance on nodosities on the roots of rootstocks derived from crosses between American species [27]. Obviously, these biotypes represent evolutionary transients generated in the process of speciation between the insect and the plant, and they are developed through diverse natural selection processes that select individuals carrying genetic variations that maximize their fitness [28].

Genetic variation in grape phylloxera populations can be caused by sexual reproduction and by somatic variation mechanisms through parthenogenetic, asexual reproduction. As stated above, its life cycle encompasses an annual sexual stage and many intervening parthenogenetic generations [1]. Compared to organisms that undergo frequent sexual recombination, the recurrent parthenogenetic generations of grape phylloxera increase its potential to reach ecological specialization in a host [29]. Sexual reproduction is reported to have generated a certain level of genetic diversity within diverse populations [30,31], but it is not clear if such events occurred in the native habitat (and subsequently were introduced into the new range as new genotypes by the secondary introduction of plant material) or in the introduced ranges [30]. On the other hand, somatic variation is considered the major force in aphid evolution [29], capable of generating novel lineages (clones) that are both genetically and phenotypically stable over time [32]. Diverse grape phylloxera clone lineages have been identified in numerous viticultural areas, indicating how edafoclimatic conditions and viticultural practices shape grape phylloxera evolution, adaptation and selection to environment [30]. In some cases, asexual reproduction has generated extremely virulent and “general purpose strains” capable of spreading, and these strains predominate in different regions and persist over time. This is the case of the G1 and G4 grape phylloxera genotypic classes (referred to as “superclones”), which were widely sampled from the root system of diverse *Vitis* spp. hosts along different Australian vineyards [33]. Genetic profiling revealed a high level of similarity between these two root-galling specialists, and a common origin by parthenogenesis from another strain was suggested [33]. Although it is expected that these generalised genotypes will be replaced in the short time by novel specialized lineages (generated by either clonal or sexual reproduction) [33], G1 and G4 lineages present a wide tolerance to a variable range of heterogeneous environments, indicating their potential to be present in vineyards over a broad geographic range.

## 2. Relevance of Studying Population Structure in Grape Phylloxera

Population genetic studies provide useful information to quantify the number of individuals present in a population. They can be used to identify the origin of the individuals in the population, and can provide an estimation of the number (and order) of the introduction events that occurred in a specific region [34,35]. As aforementioned, grape phylloxera is a major pest of worldwide grape production. Its indigenous range extends from southern Canada to northern South America, but it is more common in the eastern and central USA [18]. The diverse climatology and the large number of American *Vitis* spp. in these regions played an important role in the genetic diversity of the insect in its native range, where clear evidence of genotypes associated with host plant preference has been reported [18]. In this regard, it is of considerable interest to elucidate how the different components of the native habitat (both biotic and abiotic factors) have influenced its diversification and adaptation. Understanding the genetic and molecular determinism of existing grape phylloxera natural genetic variation will not only be useful to unravel the adaptation mechanisms of the insect to different hosts and different environments in the past, but will also provide critical information to cope with this pest in the future, especially considering new critical issues derived from climate change and novel vineyard management practices. Although such information is scarce, it has been suggested that climatic factors, notably temperature, may affect the survival and development of grape phylloxera [2,36,37], and it has been indicated that different genotypes have different optimal temperature ranges [38].

Grape phylloxera population genetics studies can provide useful insights into pest population dynamics over time and space. The natural dispersal of grape phylloxera is limited [39], but human practices have helped to spread this insect between viticultural regions [2]. Grape phylloxera populations in introduced ranges are initially genetically complex populations as the result of: (I) independent or simultaneous introductions from its native area (or other introduced ranges); and/or (II) sexual recombination and somatic mutation events that generate novel genotypes. These genotypes are then subjected to selection pressures in which the genotype better adapted to the new environment will dominate over time, reducing the overall genetic variability of the population [39,40,41]. Advances in the understanding of this complexity will aid in determination of why some of the rootstocks generated 100 years ago are still resistant to grape phylloxera effects [42]. From a practical point of view, determining the level of grape phylloxera genetic diversity in renowned wine-producing regions is essential for the breeding and selection of new grape phylloxera-resistant rootstocks and for the adequate management of the pest in infested vineyards [25]. Thus, recommendations for the particular use of a rootstock should consider the number of different biotypes and strains present in an infested vineyard [20]. In addition, this information is essential in order to set quarantine measures to avoid the incursion of exotic grape phylloxera in a region and the spread of endemic populations [43].

Furthermore, population genetics studies can be useful to evaluate the point of origin of the grape phylloxera genotypes currently present in worldwide vineyards, as well as to determine the level of admixture between overlapping populations [18]. The latter can aid in understanding the factors that influence sexual and asexual reproduction stages during the insect’s complex life cycle.

At this point, it is also worth mentioning the approaches implemented in grape phylloxera management strategies for the early detection of the insect in vineyards via DNA-based methods [44,45,46]. The development of a molecular method for grape phylloxera detection is especially relevant in grape-growing regions, where surveillance and prevention (through quarantine regulations) are the main factors for pest management [47]. However, as these methods have not been used for studying grape phylloxera population and evolutionary genetics, they will not be further discussed in this review.

## 3. Use of DNA Markers for Grape Phylloxera Genetics

Over the last three decades, insect molecular studies (including analyses of phylogeny and genetics relatedness, population dynamics and gene/genome mapping) have been boosted by the use of DNA-based molecular markers [35,48,49,50,51]. DNA markers like random amplified polymorphic DNA (RAPD), expressed sequence tags (ESTs), amplified fragment length polymorphisms (AFLPs) and restriction fragment length polymorphisms (RFLPs) were rapidly applied to the analysis of insect populations, both at small and large spatial scales [35,48,50]. Most of these markers are directly visualized as discrete bands revealed by agarose or polyacrylamide gel electrophoresis, using various chemical agents. Nevertheless, their use in population genetic and behavioural studies was replaced by the advent of high resolution polymorphic DNA markers like minisatellites and, especially, microsatellites [50]. In this regard, microsatellites have become widespread in entomological studies due to their polymorphic nature and the possibility of high throughout genotyping by automated sequencers [48,50]. Although the cost is relatively high compared to other markers (such as AFLPs or RAPDs), they are generally preferred in insect population studies, where a high number of individuals need to be genotyped for meaningful statistical tests to be performed [48]. In addition, the visualization of microsatellites can be easily achieved by the use of genetic analyzers, allowing the automation of the process by detecting the fluorescent emission of labelled primers or the fluorescently-labelled nucleotides of DNA sequences. Alternatively, the mitochondrial cytochrome *c* oxidase subunit I (COI) gene is also frequently sequenced for phylogenetic studies in insects [52].

**Figure 3:**
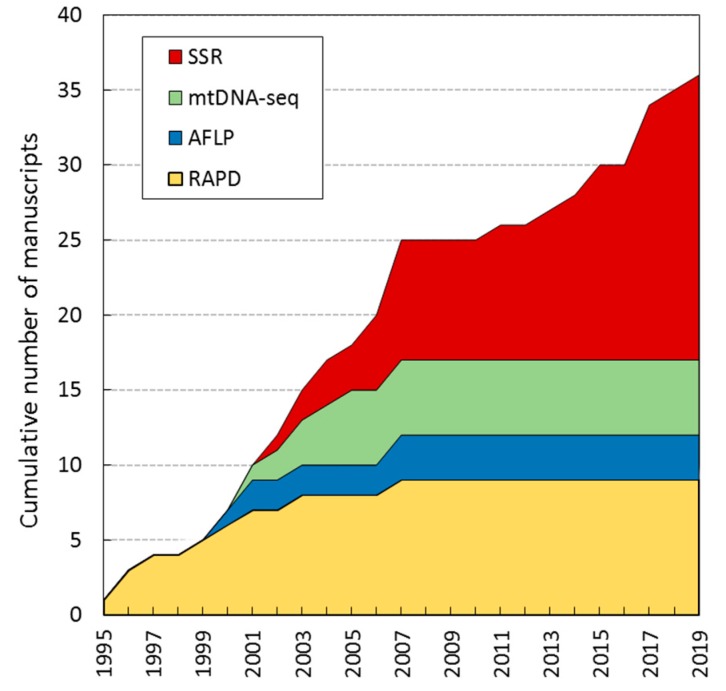
Cumulative number of manuscripts published between 1995 and 2019 reporting the use of DNA molecular markers for grape phylloxera (*Daktulosphaira vitifoliae* Fitch) genotyping. SSR: single sequence repeat; mtDNA-seq refers to cytochrome c oxidase subunit I (COI) gene sequencing; AFLP: amplified fragment length polymorphism; RAPD: random amplified polymorphic DNA.

The genetic structure and the reproductive mode of grape phylloxera have been elucidated by the use of four types of DNA molecular markers (Figure 3). Genetic variation was initially explored by the use of RAPDs in insects collected in the insect’s native range [53] and in several introduced ranges such as California [54] and Australia [20]. Thereafter, several works explored the potential of AFLPs for the analysis of grape phylloxera interclonal and *intra*clonal diversity [40,55]. An alternative characterization of grape phylloxera populations was achieved by the comparative analysis of the mitochondrial DNA COI gene sequence of several individuals isolated from North America [24,56], Australia [25], and South Africa [21]. The availability of more efficient molecular tools and new sequencing technologies allowed the detection of simple sequence repeats (SSRs) in the grape phylloxera genome. As a result, diverse sets of flanking primers were developed for the establishment of high-throughput microsatellite genotyping procedures [33], which were adopted by diverse laboratories as a reference technique. The high specificity of this type of marker, together with their high reproducibility (which allows comparison of results among laboratories) rapidly replaced previous genotyping systems, and nowadays it is the most common system for grape phylloxera genotyping. In fact, it is the only approach with a growing trend in the last decade (Figure 3). By this means, studies have involved analyzing the genetic structure of grape phylloxera using microsatellites in its natural habitat [18] and in many introduced ranges [17,19,31,39,57,58,59].

### 3.1. RAPDs and AFLPs

The random amplified polymorphic DNA (RAPD) technique was the first genetic tool used in grape phylloxera population and evolutionary genetics studies [54]. This technique is based on the amplification and analysis of random regions of the genome of the organism in question, without requiring prior knowledge of its structure or sequence data. RAPDs are generated by applying the polymerase chain reaction (PCR) to genomic DNA samples, using randomly designed oligonucleotides as primers. As a result, multiple amplified fragments of different size (corresponding to different loci) are obtained, which can be separated and scored on a standard agarose gel [60]. This technique was firstly used to analyze the genetic diversity of 13 grape phylloxera populations from California, selected to represent diverse grape phylloxera biotypes [54]. Results indicated an unexpected high level of genetic diversity, revealing as much diversity between populations as within biotypes, suggesting the existence of multiple introductions, strong selective pressure from different rootstock hosts, or excessively high mutation rates. In addition, this study was useful when speculating about an undetected sexual cycle in the life cycle of the insect, considered as parthenogenetic at the time [54]. Soon afterwards, Lin et al. [53] analyzed the genetic structure and diversity of grape phylloxera populations growing on *V. arizonica* and *V. riparia* from two locations of its native range (central Arizona and New York, respectively). After a screening to select a set of RAPD primers capable of generating reproducible results, these populations were genotyped by 12 RAPDs, generating up to 108 bands ranging from 0.2 to 2.5 kb in size. The different banding patterns obtained between both sites highlighted for the first time the likely effect of geographic distance, host and abiotic conditions on grape phylloxera population structure. Similarly, a comparative analysis of six grape phylloxera populations from Australia was performed using nine RAPD primers [20]. This set of primers generated 71 bands, of which 47 were polymorphic, and it was possible to cluster the samples into three distinct genetic groups, indicating the likely introduction of different strains into that region, and/or a high level of sexual recombination or somatic variation within the introduced populations. A high degree of genetic polymorphism was also observed following the use of 13 RAPD primers to genotype 49 grape phylloxera individuals collected from *V. vulpina* and *V. aestivalis* vines in central Missouri [61]. Following this work, most of the amplified fragments ranged in size from 0.4–1.1 kb, and a total of 93 bands (48 polymorphic) were scored. Analysis revealed that ca. 6.5% in RAPD banding variability was due to the host species, whereas ca. 8.0% was attributable to spatial variation. The genetic diversity of grape phylloxera was also evaluated in Southern France through the use of 15 RAPD primers [22]. Although a high diversity was found within the 20 samples collected from leaf galls, the genetic structure of this population did not seem to be influenced by the plant host, habitat or location, suggesting that the new strains generated by either sexual events or somatic mutations possessed the ability to move between regions and between host genotypes.

Another genotyping method, amplified fragment length polymorphisms (AFLPs), has been successfully applied for grape phylloxera studies. As in the case of RAPDs, the utility of the AFLP method lies in that it does not require prior knowledge of an organism’s genome. This method uses restriction enzymes coupled with PCR to generate many hundreds of unique fragments (generally visualized as bands by gel electrophoresis) that can be used to genotype individuals within or among populations [62,63]. AFLPs were firstly used to analyze the genetic structure of grape phylloxera populations in Europe, considering 103 populations from 7 geographic regions [55]. The analysis of the 479 amplified fragments (obtained using 9 primer combinations) revealed a grouping into two clusters weakly associated with collection site location, and the genetic structure of the European grape phylloxera was not affected by plant host. Later on, this technique was coupled to DNA sequencing for the detection of genetic diversity within single founder lineages of grape phylloxera, using 8 different clonal lineages and a minimum of 15 parthenogenetic generations [40]. Following this work, the approach was useful in detecting genetic variation in all lineages from early generations, with up to 15 somatic variations being detected per lineage (most of them in noncoding regions), which accounted for up to *ca*. 4% of the total amount of genetic variation.

As observed, both techniques proved to be inexpensive yet useful grape phylloxera genotyping methods, and they attracted widespread interest for grape phylloxera population and evolutionary genetics studies. Nevertheless, they have the main disadvantage that nearly all RAPD and AFLP markers are dominant [62,64] and it is not possible to differentiate if the amplified fragment (visualized as bands on a gel) correspond to a locus that is heterozygous or homozygous. Thus, amplification products are exclusively scored as present or absent [33,55], which reduces the amount of information potentially obtained and hinders the use of certain statistical analyses. In addition, reproducibility can be a problem, especially in the presence of weakly amplified bands [64].

### 3.2. Cytochrome C Oxidase Subunit I (COI) Gene Sequencing

Other types of markers used for molecular studies are those derived from the direct sequencing of targeted DNA regions. In contrast to AFLPs or RAPDs, these methods require precise knowledge of the region of the genome of interest. Nevertheless, once amplified by PCR through the use of specific primers, and sequenced, it provides the most detailed level of information for genetic analyses. Thus, DNA sequencing is ideal to inspect the evolutionary history of an organism and for inferring evolutionary processes involved in the migration and expansion of an animal species. Considering that it is easy to isolate and amplify, and that it is highly variable in natural populations, mitochondrial DNA (mtDNA) is of special interest for molecular diversity studies [65]. Compared with other mitochondrial genes, the sequence of the cytochrome *c* oxidase subunit I (COI) possesses a high incidence of base substitutions that leads to a high rate of molecular diversity, creating signals that might reflect population history events. Consequently, the comparative analysis of the COI gene sequence allows not only the discrimination between closely related species [66], but also between phylogeographic groups within a single species [67,68]. In addition, the ability to use universal primers for fragment amplification (and further sequencing) enables the easy and efficient recovery of molecular information from most animal phyla at an affordable cost [69]. As a result, the COI gene sequence is regularly used as a global DNA-based identification system for animals, providing useful information on the diversification and evolution patterns of numerous species [70]. As for many species, this gene has being used to examine the evolutionary rate (and its implications for evolutionary purposes) in insects species like the meadow grasshopper (*Chorthippus parallelus*) [52] and the house fly (*Musca domestica*) [71], or, more recently, the cotton aphid (*Aphis gossypii*) [67] and the stingless bee *Partamona rustica* [68].

The analysis of the COI gene sequence has provided valuable insights into the evolution pattern of grape phylloxera in different regions. A high level of genetic diversity and phylogeographic structure was found by Downie et al. [24] through the analysis of a 473 bp fragment of the COI gene sequence in 89 grape phylloxera individuals sampled from wild vines in 24 states of the USA. Among other findings, the haplotype analysis of the COI gene sequence suggested the presence of at least two independent sources of introduction of the insect into the vineyards of the Pacific Coast of the USA. Whereas all the samples collected in California seemed to be closely related to grape phylloxera genotypes isolated from *V. vulpina* plants from the Atlantic coast, samples from northern regions of the states of Oregon and Washington resembled individuals collected from *V. riparia* plants in the northeast USA. A similar approach was used to evaluate grape phylloxera population dynamics in the southwest USA (12 populations in Arizona and New Mexico) [56]. Results revealed the presence of significant stratification at a geographic level, suggesting an early split of the insect population in this region, relatively isolated during vegetational expansion events.

Following this work, the authors suggested that this stratification could have been overlaid by the recent introduction(s) of new genotypes from midwestern regions. The high variability of the COI gene sequence was also useful in drawing important conclusions on the origin and pattern of introductions of grape phylloxera into worldwide viticultural regions [12]. The comparative analysis of this sequence in samples collected from the native and introduced ranges suggested that two widely divergent lineages originating in different geographical regions and on different hosts were inadvertently introduced into global viticulture. These two lineages probably originated on infested *V. riparia* vines in the northeast USA, and they could have been introduced simultaneously into worldwide vineyards during the movement of plant material in the late 19th century. On the other hand, gene genealogy supported the widely accepted assumption that grape phylloxera was predominantly introduced into Europe via France [10], although additional minor independent introductions may have occurred in specific regions. Furthermore, it was suggested that grape phylloxera strains present in Peru, Australia and New Zealand were originally introduced from plants transported from California [12], confirming this region as a hotspot for the worldwide dispersal of grape phylloxera.

The pattern of introductions into Australian vineyards was further analyzed by Corrie et al. [25]. Among the 14 genotypic classes of grape phylloxera examined (representing 334 insects collected in diverse regions), three mitochondrial haplotypes were identified based on the variation detected at 20 loci within the COI gene sequence. The analysis of these classes revealed the presence of two clades, which partially correlated with vine host usage. These findings indicated that different lineages of grape phylloxera were introduced into Australia with different hosts; one closely related to Californian genotypes, and another with a high similitude to strains isolated in Oregon, Washington, Europe, Argentina and New Zealand. How grape phylloxera reached South African vineyards was also explored by the analysis of the COI gene sequence [21]. After the sampling of grape phylloxera populations from three contrasting regions (Orange River, Olifants River and Western Cape), the authors suggested that contrary to previous findings [12], grape phylloxera in South Africa is not the result of a single introduction, but of at least two independent events. Following this work, the strong differentiation of one of the grape phylloxera haplotypes from those inferred from insects collected from other introduced ranges indicates the presence of one direct introduction from the native range.

These studies highlight the usefulness of the COI gene sequence to trace the evolutionary dynamics of grape phylloxera in its native range, and they have been very informative for elucidating the origin of genotypes accidentally introduced into new habitats. However, it has also been shown that differences among nearby populations can exist, but they can be too recent to be detected with the mere analysis of mtDNA sequences [56]. In addition, Corrie and Hoffmann [39] indicated that mtDNA haplotypes cannot be used to predict the feeding ability of grape phylloxera lineages. Thus, the use of a single mitochondrial gene sequence seems to be insufficient to resolve complex relationships among grape phylloxera populations [24], and thus their analysis will benefit from an integrative approach combining nuclear and mitochondrial genes (as well as morphological characters and ecological information, if available) to trace the ancestry of the insect in a global context.

### 3.3. Microsatellites

Microsatellite markers (or single sequence repeats, SSRs) are polymorphic DNA loci containing a unit of repetition (or motif, such as “CT” or “TAA”), which typically comprises between two and eight base pairs [72]. In contrast to single nucleotide polymorphisms (SNPs), a microsatellite generates alleles of varying length, as the number of repeats for a specific locus differs between individuals [73]. Microsatellites are among the most variable types of DNA polymorphism in the genome, being especially suited to linkage mapping and association studies, and the genetic identification of individuals within a population. Some of the reasons explaining their popularity in population studies are their abundance in the genome, as well as their high level of polymorphism (derived from the high variability in the unit of repetition), their reproducibility and their codominant nature [74,75]. Since the early 2000s, nuclear microsatellites genotyping rapidly replaced RAPDs, AFLPs and mtDNA sequencing approaches in studies dealing with grape phylloxera ecology and evolution (Figure 3). The availability of multiplexing many PCR products in a single run, together with the use of automatic sequencers and the use of algorithms for automated allele sizing and characterization, eased the high throughput screening of a large number of loci in a fast and precise way. As a result, microsatellites have become the markers of preference in grape phylloxera population genetics studies and related areas [42,76]. Nonetheless, the development of a reliable marker can be a costly and iterative process that involves time-consuming cloning steps that rarely can be parallelized [72]. The first set of microsatellite markers for grape phylloxera genotyping (*Dvit1*–*Dvit4*) was developed by Corrie et al. [33] (Table 2). These four loci were characterized in 361 individuals from 28 vineyards, and they provided between three and seven alleles per locus, differentiating up to 45 different genetic profiles (or multilocus genotypes) in the test set. This set of markers was further extended by Vorweck and Forneck [30], by adding the *Dvit5* and *Dvit6* loci to the previous dataset (Table 2).

Taking advantage of recent advances in sequencing technologies, Lin et al. [42] explored the grape phylloxera genome to identify alternative microsatellite loci with high levels of sensitiveness and effectiveness. Following a subtractive-based hybridization strategy, up to 50 microsatellite loci were identified for primer design, 19 of which produced clear PCR products. After their evaluation in 32 grape phylloxera samples from California and Europe, *DVSSR3*, *DVSSR4*, *DVSSR6*, *DVSSR7*, *DVSSR9*, *DVSSR16* and *DVSSR17* primers reliably detected between two and eight alleles per locus (Table 2), resulting in a suitable dataset for the large-scale genetic analyses of grape phylloxera populations. Aware that the number of markers available was limited, and that most of them generated only two to four alleles per locus, Riaz et al. [76] analyzed an alternative set of 89 new microsatellite markers (from an initial screening of 130) for grape phylloxera genotyping. This large dataset was initially evaluated considering factors like their polymorphic nature, reproducibility, and the absence of null alleles. After their rating, a reduced final set of 28 microsatellite markers was proposed for analyzing grape phylloxera genetic diversity (Table 2). Although these two works expanded the ability of characterizing grape phylloxera at a genetic level, the genotyping of such a large number of loci is not realistic from a practical (and economical) point of view.

As observed, different sets of microsatellite markers are available for grape phylloxera genotyping, but the grape phylloxera scientific community will benefit from a harmonized set of markers and the establishment of some general rules for their use in the identification of different strains. In an effort to standardize methodology, the ISHS Phylloxera Working Group suggested the use of 10 robust microsatellite markers for grape phylloxera genotyping (Table 2), and proposed a set of reference alleles from a series of reference biotypes [79]. This core set of primers was obtained from published [30,33,42,76] and unpublished datasets, and it was selected considering parameters like (I) allele size, (II) allele frequency, (III) technical robustness for amplification and scoring, and (IV) suitability for multiplexing [79]. This set of primers was tested in a sample of 103 individuals collected from different regions, identifying 63 different multilocus genotypes. According to the authors, the use of a reduced number of markers can identify a high number of the multilocus genotypes present in a sample, which will considerably reduce operational costs. Accordingly, seven of these markers (*Dvit6*, *DVSSR4*, *DV4*, *DV8*, *Phy_III_55*, *Phy_III_30*, and *Phy_III_36*) proved to be informative enough for the identification of 203 multilocus genotypes from 335 grape phylloxera individuals collected from southern Germany and Switzerland [31]. Obviously, the rate of identification depends on which markers are combined, so further studies testing this core set of microsatellites in wider samples, considering both the native and the introduced ranges, are needed to select the optimum number (and combination) of primers.

As mentioned above, microsatellites are now the markers of preference in grape phylloxera genetics studies, and many reports have been published analyzing the population structure of the insect both in its native range and in different introduced regions (Table 3). The population structure of the insect across its native range was recently analyzed by the use of 32 microsatellite markers in more than 500 insects from 19 states of the USA [18]. Clustering analyses revealed the existence of a strong genetic structure that linked grape phylloxera diversity to host plant and geography, although the latter could be the result of the non-random distribution of the *Vitis* species in the native range of grape phylloxera. Thus, five populations distinguished by their *Vitis* host and geographic location were indicated: (I) *V. vulpina*-east population; (II) *V. vulpina*-west population; (III) *V. riparia* population; (IV) *V. arizonica* population; and (V) *V. cinerea* population. In addition, reproductive statistics together with the presence of a high number of admixed samples confirmed that sexual reproduction is a common mechanism in the native range to create new genotypes of grape phylloxera.

Fifteen microsatellite markers were used to elucidate the population diversity of grape phylloxera in the introduced range of California [19]. The analysis of a large collection of leaf and root forms of grape phylloxera collected from different locations indicated the presence of four genetically distinct subpopulations in California, including a recently introduced subpopulation consisting of exclusively foliar-feeding forms. This subpopulation was confirmed later to be the result of a mixture of the *V. riparia* and *V. vulpina*-west populations, probably originally introduced from the state of Indiana, where both *Vitis* species overlap [18]. Besides, the analysis of the other three subpopulations (mainly formed by root-feeding forms) suggested some level of adaptation of grape phylloxera to specific rootstocks, as well as enough evidence to confirm sexual reproduction events between subpopulations.

The genetic structure of grape phylloxera populations in Europe was firstly analyzed using six microsatellite markers [30]. The screening of these loci in 360 insects from six viticultural sites in France, Germany and Spain revealed the presence of 195 unique genetic profiles. Following this work, no overlapping strains between sampling sites were observed, indicating the presence of site-specific populations and providing evidence that parthenogenesis is the predominant mode of reproduction of grape phylloxera in Europe. The latter view was supported by several studies performed on leaf-feeding grape phylloxera in diverse Austrian, German and Swiss viticultural regions [31,58], although some signs of sexual reproduction events were observed too. These two works highlight the high diversity of the European populations, as well as the absence of a “superclone” dominating all over the continent. In addition, the joint analysis of European genotypes [31] with those from the native range [18] supported the notion that the grape phylloxera strains present nowadays in Europe are likely the result of introductions of plant material from northeastern America, where *V. riparia* dominates.

By the screening of the four microsatellite loci initially identified by Corrie et al. [33], some insights into the genetic structure of grape phylloxera and host-associated clones in Australia were apparent [25,33]. Genotypic patterns of grape phylloxera analyzed in four locations supported the hypothesis that root populations are comprised primarily of obligate and/or functionally parthenogenetic clonal lineages, and the temporal persistence of one multi locus genotype in both the root and leaf galling populations and on multiple leaf galling plants suggested the clonal spread between leaves and roots on the same plant, as well as the successful overwintering of parthenogenetic insects. Furthermore, the high genetic diversity found for the isolates obtained in the Rutherglen area (Victoria state) confirms historical reports that indicate that this viticultural area received extensive introductions of *Vitis* material from Europe, which unintentionally could have introduced multiple grape phylloxera lineages [77].

Microsatellites have also been useful in exploring the genetic diversity of grape phylloxera in other introduced regions in Uruguay [57], Argentina [17] and China [80]. Regarding Uruguay, the cluster analysis of 69 leaf-galling individuals from six viticultural regions using four microsatellite markers (*DVSS3*, *DVSSR4*, *Dvit1* and *Dvit2*) revealed a stratification that correlated with geography, as grape phylloxera samples collected from nearby regions grouped together. In addition, the joint analysis of Uruguayan individuals with foreign grape phylloxera populations (from Brazil, Peru and Europe) discarded the introduction of grape phylloxera strains from Peru (which are suggested to come from California [17]), and supported the European origin of local genotypes [57]. In Argentina, the analysis of 129 samples from four provinces (Mendoza, San Juan, La Rioja and Río Negro) using 21 microsatellite markers revealed a high level of diversity, identifying up to 17 different multilocus genotypes [17]. The hierarchical cluster analysis showed two major Argentinean grape phylloxera groups that could reflect two initial independent introductions that spread over the country and adapted (by either sexual or asexual reproduction events) to different environmental conditions. Lastly, the analysis of 31 individuals from four regions in China (Shanghai, Hunan, Shaanxi and Liaoning) revealed a general low genetic variation, and no association between grape phylloxera and host variety was found. Nevertheless, cluster analyses revealed that the genetic profiles could be split into two groups that correlate with sampling site, indicating the existence of at least two independent introductions into China [80].

As codominant and highly variable DNA markers, microsatellites have proven to be a powerful tool for unravelling part of the evolutionary history of grape phylloxera in both its native and introduced ranges, and they have been particularly useful to prove the occurrence of sexual recombination events capable of generating new genotypes of grape phylloxera. Despite many advantages, microsatellite markers also have pitfalls (e.g., large allele dropouts, shutter due to slip strand mispairing during PCR, null alleles due to mutations in priming sites, and polyploids) that can impact the ability to draw sound conclusions from SSR marker data and limit their utility in ecological studies [82]. Fortunately, many of these drawbacks can be avoided if an appropriate selection of loci is achieved [83]. Nevertheless, the size-based scoring of the alleles assumes that all distinct alleles differ in length, but alleles of the same length but different origin can appear [84]. This phenomenon (known as homoplasy) reduces the visible allelic diversity of a population, and it can be problematic for the reconstruction of phylogenetic relationships and thus elucidation of diversification and evolution patterns [83].

## 4. What Is Next?

As reviewed in this article, grape phylloxera population genetics has been sustained for 25 years by the analysis of microsatellites and mtDNA, as well as anonymous markers like RAPDs and AFLPs. The grape phylloxera scientific community rapidly reacted to fulfil the requirements of molecular ecologists and evolutionary biologists in terms of analytical and computational needs. In recent years, the advent of new technologies demands new adaptation processes if we wish to increase our knowledge on grape phylloxera population genetics. Recent technological advances in DNA and RNA sequencing have opened a plethora of possibilities to rapidly generate large-scale sequencing data suitable for ecological studies. In this context, next generation sequencing (NGS) technologies allow the massive identification of genetic polymorphisms (mostly SNPs), even when only a small fraction of the genome is sequenced [85]. When multiple sequences of a genomic region are generated from different individuals of the same species, genetic variations can be detected, providing an ideal basis for phylogenetic reconstruction if a broad section of the genome is covered. The utility of NGS for insect ecology studies has been widely demonstrated [86,87,88]. As an example, more than 490,000 genetic polymorphisms were discovered in the diamondback moth (*Plutella xylostella*) by the restriction-site-associated DNA sequencing (RAD-Seq) of nine populations collected from Australia [87].

Consequently, one of the most interesting future aspects for grape phylloxera population and evolutionary genetics studies is the application of high-throughput genotyping technologies for SNP discovery. SNPs are abundant in insect genomes [89], and it is likely that it will be likewise proven to be so in the case of grape phylloxera. Although not sequenced yet, initiatives are underway to fully sequence its genome [90], which will be useful for the efficient and genome-wide detection of SNPs. The abundance of SNPs and their genome-wide distribution has enormous applications in insect genetics, most especially for population genetics studies [89]. Compared to microsatellites, SNPs present many advantages and, as they represent unique mutation events, they are less prone to errors when reconstructing phylogenetic relationships between haplotypes [91]. Considering their biallelic nature, a larger number of SNPs need to be screened to obtain the statistical power given by a standard set of multiallelic microsatellites for the appropriate assessment of population structure [92]. Nevertheless, large SNP datasets can be obtained by NGS technologies with the budget commonly associated with microsatellite genotyping. In fact, comparative analyses have shown that denser sets of genome-wide SNPs are preferable than a reduced set of microsatellites for a precise estimation of population structure [93]. In addition, biallelic SNPs can be efficiently combined as multiallelic markers by reconstructing local haplotypes, generating highly informative markers for population genetics and overcoming the limitations linked to the biallelic nature of SNPs [94]. Thus, a possible future step for grape phylloxera population genetics is the high-throughput genotyping of a large number of individuals by genotyping-by-sequencing (GBS). This approach allows the simultaneous discovery and genotyping of a large number of SNPs in a single step without the need for a reference genome sequence [95]. As shown in different species [88,96], GBS is perfectly viable for the high-throughput genotyping of insects. As an example, more than 23,000 SNPs were detected by GBS in the genome sequence of 93 individuals of the oriental fruit moth (*Grapholita molesta*), which were then used to accurately infer the genetic structure and evolution patterns of this insect in Brazil [88]. An alternative approach is the generation of a SNP microarray for grape phylloxera genotyping. With this aim, candidate SNPs could be identified through the sequencing of a small set of highly different individuals (ideally representing the different biotypes indicated in [27]). Then, sequence information for highly informative SNPs could be used to design specific probes than can be integrated in a customized microarray for the rapid and large-scale genotyping of grape phylloxera populations.

As reported for the model species *Drosophila melanogaster* [97,98], a further advantage of the detection of a massive number of genetic variants by NGS is the possibility of association genetics studies in grape phylloxera, in which specific genetic variations are statistically associated with complex phenotypic traits. As mentioned above, up to seven grape phylloxera biotypes have been differentiated [27], but the complex genetic basis of this differential behavior has yet to be elucidated. Thus, association genetics studies (such us genome-wide association studies, GWAS) could reveal relevant genomic regions and alleles that favoured the specialization of different lineages on different hosts as a result of the grape phylloxera-grapevine coevolutionary history, information that should be considered when developing new resistant rootstocks.

On the other hand, novel –omics technologies also open new opportunities to advance our understanding of grape phylloxera natural diversity. Recently, RNA libraries of the two forms of the grape phylloxera (leaf-feeding and root-feeding forms) have been sequenced to perform a *de novo* assembly of its transcriptome to identify the mechanisms that are responsible for this differential feeding behaviour [99]. Following this work, a catalogue of more 105,697 transcripts has been created, for which 17,372 proteins (12,617 of them complete predicted proteins) were successfully predicted. Furthermore, the upcoming availability of a reference genome [90] will improve our understanding of grape phylloxera genome organization. Preliminary data reveals that the grape phylloxera haploid genome is approximately 400.5 Mb long [100], which is arranged in 5 chromosomes (2n = 10) [101]. Its complete sequencing will provide basic information for genetics studies, including the total number of genes in the genome, the presence of duplication events, or the relative amount of repetitive events. Its publication will provide a new research framework for approaches like genetic mapping, genetic identification or genetic diversity studies, and it will be useful to solve recurrent issues in grape phylloxera genotyping. As an example, it is common to find more than two alleles in diverse microsatellite loci in different strains [18]. The availability of the reference genome will clarify if the presence of additional alleles is because of a technical problem (low specificity of primer sequences) or if there is an actual biological reason, and the genomic region flanked by such primers is truly duplicated in the genome as a result of evolution and adaptation mechanisms.

## 5. Conclusions

Undoubtedly, the development of DNA-based molecular markers for grape phylloxera genotyping has been one of the major advances in the grape phylloxera scientific community in the last 25 years. The establishment of efficient procedures for data acquisition and data analysis has increased our knowledge of the interaction between this pest and *Vitis* spp., allowing the analysis of its ecology in its native range and in introduced regions worldwide. However, significant questions and challenges remain that need to be addressed. Information regarding grape phylloxera genetic diversity within and among the native and introduced ranges is not completely clear, and how extremely virulent grape phylloxera strains emerged in diverse introduced viticultural regions (Australia, California) is still not fully understood. In addition, our current knowledge of the effect of future climate change scenarios on phylloxera genetic diversity should be analyzed in detail for efficient future management strategies for this pest to be designed and implemented in the vineyard.

In this light, there are many intrinsic factors derived from the biology and ecology of this insect that need to be considered in the designing of future population and evolutionary genetics studies. Research will benefit from a standard protocol for the collection of both root-feeding and leaf-feeding forms, as otherwise the results obtained may be biased. As an example, leaf-feeding grape phylloxera individuals hatch and form clusters in close grapevine leaves, and their growth is affected by environmental conditions and viticultural practices. Furthermore, grape phylloxera populations are commonly analyzed using classical population genetic parameters, obviating the fact that grape phylloxera is an obligate sessile biotroph on *Vitis* spp. with a limited dispersal capability. This fact might be causing potential errors in the interpretation of such metrics, hindering the appropriate analysis and understanding of grape phylloxera population dynamics.

## Figures and Tables

**Table 1:** Biotype differentiation by feeding sites, feeding organ (galled tissue), and insect development. A capital letter indicates superior insect development in compared host plants, small letters less/limited insect development, – indicates neither gall or insect development, and ? indicates no information is available (t: tuberosities; n: nodosities; p: pseudotuberosities; and g: leaf galls). Table is reproduced with permission from [27]. © American Society for Enology and Viticulture. AJEV 67:371-376.

Biotype/ Feeding Tissue	*Vitis vinifera*			Rootstocks and Hybrids (*V. vin*. x American *Vitis* species)			Rootstocks (American *Vitis* species)		
**A**									
Root	T	N	-	t	n	-	-	n	-
Leaves		-			G			G	
**B**									
Root	t	n		T	N		-	n	-
Leaves		-			-			-	
**C**									
Root	-	n	-	T	N	P	-	N	P
Leaves		-			G			G	
**D**									
Root	t	n	-	-	N	-	-	N	-
Leaves		-			?			G	
**E**									
Root	T	N	-	T	N	?	T	N	P
Leaves		-			-			-	
**F**									
Root	T	N	-	?	?	?	?	n	?
Leaves		G			?			G	
**G**									
Root	?	?	?	?	N	?	-	N	?
Leaves		G			G			G	

**Table 2:** List of microsatellite markers (SSRs) most commonly used for grape phylloxera genotyping in population and evolutionary genetics studies.

SSR ^1^	Motif	Flanking Primer Sequences (5′→3′)	Source	Reference
Dvit1	(CA)_n_ (CG)_n_	F: CGTTCGTTCTGGTATCGTTATT R: TAACGACCCGACTGAAATGTAG	[33]	[17,25,30,33,39,40,57,58,77,78]
Dvit2	(CT)_n_ (AT)_n_	F: GCTTAATTTTGTGTCTCAAGTTA R: TAATGCTTCGTTTTCTAAGTGC	[33]	[25,30,33,39,40,57,58,77,78]
**Dvit3**	(AT)_n_ (GT)_n_	F: CCAAAACAACCAAGATTTTCTCC R: GATCCAAACTATGACAAACACCC	[33]	[19,25,30,33,39,40,58,77,78,79]
Dvit4	(AAT)_n_	F: TCTTCAAAAATGTTACATGAT R: TATACAATGAATGGTATCAATTC	[33]	[19,25,30,33,39,40,58,77,80]
Dvit5	(A)_n_	F: GAAATCCGTTCGGTGAGAGC R: TATGGTCAATGGTCAATCCGTC	[30]	[30,40,58,77]
**Dvit6**	(AAT)_n_	F: TGGACGATGGTTTTCATAGC R: TTGATTGTCATTGGTTTTGC	[30]	[19,30,31,40,58,77,79]
DVSSR1	(CA)_n_	F CGGCGACGAGTTAAACTATC R TCGTTGTATAGATCTGTGTTGC	[42]	[42,80]
DVSSR2	(CA)_n_	F TCGCTACTACCAGCCGATCAG R TGAACAATGAAAGCCCTGGTGG	[42]	[42,80]
DVSSR3	(CA)_n_	F: AGCATGTGAGGTGCAAGGC R: CCTCGGGCGGAACAATCG	[42]	[19,42,57]
**DVSSR4**	(CT)_n_	F: TGGTATTCACCTTGGAGCCTAG R: GCTACTGAAACCCCTTCAACAC	[42]	[19,31,42,57,78,79,80]
DVSSR6	(CTT)_n_	F: GTTTACTGAAATAAGGGCTGG R: AGTTGTGATTATAAGCCGAGG	[42]	[19,42,78]
DVSSR7	(GCA)_n_	F: GTGAGTTGACTGTTGATTCG R: CGCAATTCATTCGGTTACC	[42]	[19,42,78]
DVSSR9	(GCA)_n_	F: CGCAATTCATTCGGTTACC R: GTGAGTTGACTGTTGATTCG	[42]	[42,80]
DVSSR16	(A)_n_	F: AGACCAGACGCGAGCAATG R: ACCATCAATGAAAGCCTTGTCG	[42]	[42,78,80]
DVSSR17	(CGTTTCTG)_n_	F: CTCTGTGTAGCCAAGTCAAC R: TATCCTACGCCAGTAAGAAG	[42]	[19,42,78,80]
**DV4**	(GTT)_n_	*unpublished*	-	[31,79]
**DV8**	(TG)_n_	*unpublished*	-	[31,79]
**DV11**	(CT)_n_	*unpublished*	-	[79]
PhyII_6 ^2^	(TA)_n_	F: TTATTGTCAGTTAGGTCTGAGATACC R: ATTGTTTTCGACCCGTCATTAT	[76]	[17,18,76]
PhyII_10 ^2^	(AT)_n_	F: CCTTCTCACTTCACATCAAAGC R: TCCAAAAGCTATATGATCCCCTA	[76]	[17,18,76]
PhyII_13 ^2^	(AC)_n_	F: GCGTATAAACGATGGCGTTAAA R: TCTTCTTCACGTTTGCTCAGAA	[76]	[17,18,76]
PhyII_16 ^2^	(AT)_n_	F: CTGGTGGCTTTGGTGGTAAG R: CTCGATCTTGCCTGCTACCTAT	[76]	[17,18,19,76]
PhyII_23 ^2^	(AT)_n_	F: CGTATGCCCTTCTAACACGATT R: CGGGATATTCGATTAAATGCTG	[76]	[17,18,19,76]
PhyII_26 ^2^	(AT)_n_	F: TTACTATTTGGCCGTCAAGTCA R: GCTGAAAGAGCAACAAATTCAA	[76]	[76]
PhyII_28 ^2^	(AT)_n_	F: CCGAGAGCAAGAGAAAACTGAG R: TCGTACATTCAAGTTACTTTTACACA	[76]	[76]
PhyII_29 ^2^	(AT)_n_	F: CCAATCATTTTACTAGGCTCGTG R: GAGGCGATAGCAGAGTATGGAG	[76]	[76]
PhyII_31 ^2^	(TG)_n_	F: CGTCGCCCTTATATCAAATTCT R: GCGGTGATGGACTGTAGAAAAT	[76]	[17,18,76]
PhyII_32 ^2^	(GT)_n_	F: ACGTATTAATGGGCGTCGTTAT R: TTAAAATATTGCCGCAAGTTCA	[76]	[19,76]
PhyII_34 ^2^	(AC)_n_	F: AAGCCGGTCTGCAATATTATGT R: TTTCGTTTACACAAGAATGGTATG	[76]	[17,18,76]
PhyII_36 ^2^	(AC)_n_	F: CGTACCCCACACAGAGTATTCA R: CCCTCATACACTCACACTCGAA	[76]	[17,18,76]
PhyIII_15 ^2^	(TGT)_n_	F: TTCCAGTAGTTGCTGTTATTCCTG R: AACCACAGAATTTTCCTTTTGTTC	[76]	[17,18,76]
PhyIII_19 ^2^	(ATT)_n_	F: CGCCGATTTATGTATCAACTCA R: GACTGTTTCGTACCGCACATAA	[76]	[18,76]
**PhyIII_30** ^2^	(TCT)_n_	F: ACCGTTATGAACAAAAGCAGGA R: GGTTTTGCCTTCAGACTCCTT	[76]	[17,18,31,76,79]
**PhyIII_36** ^2^	(TAA)_n_	F: CGTCCTTCTTGCGTGATATTTT R: GGCGGAATAAATGAGAAAAGTG	[76]	[18,19,31,76,79]
PhyIII_38 ^2^	(GAA)_n_ (GAC)_n_	F: TTGATGAAAATGCTCCTTGTTTT R: CTGGTGGTTCAGTATTCTCGTC	[76]	[76]
PhyIII_42 ^2^	(TA)_n_ (CGG)_n_	F: GTATATACGGTGGCGGTAGGAC R: CGTACTCAAGTCGCTATACCCTA	[76]	[17,18,76]
PhyIII_46 ^2^	(CCA)_n_	F: TCTCGCACGGCTATTGTAGTTA R: TCTGTTGCAATGCCTAAAAGAA	[76]	[17,18,76]
PhyIII_49 ^2^	(TAA)_n_	F: CCATCTTAAATCTTTGGCTCGT R: ACGGAACTACACACGCACATAC	[76]	[17,18,76]
PhyIII_53 ^2^	(ATA)_n_	F: CACTCATGATTGCAATTTTTCC R: TTGCACATAGTGTGATACATTTCC	[76]	[17,18,76]
**PhyIII_55** ^2^	(ATT)_n_	F: CGTATGATCGTCACAGAGGAAA R: CGATTCCGCTTTAAACAATACC	[76]	[17,18,31,76,79]
PhyIII_61 ^2^	(ATA)_n_	F: GTACCGGCCGAAAATTGTATT R: ACCTCCACCTAAGCGAGAAAC	[76]	[17,18,19,76]
PhyIII_63 ^2^	(AGC)_n_	F: GTGTGGTAATTTATGGGCGTTT R: CAAAGTGGGCACGATAAAATTG	[76]	[17,18,76]
PhyIII_65 ^2^	(ATT)_n_	F: TTTACTATCATAGCTTTCCACTTGAAC R: GGGTATTTTTGGGTTTAATTCTACTG	[76]	[17,18,76]
PhyIII_69 ^2^	(TAA)_n_ (ATT)_n_	F: CTTTCTCTCCCGATTGTCCTT R: GGCCTTTAACGCGTAGGTAGAC	[76]	[18,19,76]
P hyIII_86 ^2^	(TAT)_n_	F: AACAAAGTCCACTTTCGCTGTT R: CACGGTCTGCATAAATCACTGT	[76]	[76]
PhyIII_87 ^2^	(ATT)_n_	F: TTCAGAATCGACGTCAGCTAAT R: CATTCGACTCTAGCAATACCAAA	[76]	[17,18,76]
PhyIV_4 ^2^	(AATA)_n_	F: CAGGCATCTCAAATGGATTAGC R: TGCGTCATTTCATTAACTTACACTT	[76]	[17,18,19,76,79]

^1^ Microsatellite markers adopted by the ISHS Phylloxera Working Group [79] as reference are indicated in bold. ^2^ Primer sequences were retrieved from the NCBI database. SSR motifs were identified using the Simple Sequence Repeat Identification Tool (SSRIT) implemented in [81] (accessed on 01 July 2019).

**Table 3:** Main studies aimed to explore the genetic diversity and structure of grape phylloxera populations in diverse regions by means of microsatellite markers (SSRs).

Region	SSRs (n)	Samples (n)	Multilocus Genotypes (n)	Main Genetic Groups (n)	Ref.
Native range	32	549	466	5	[18]
California	15	296	145	4	[19]
Europe	6	360	195	-	[30]
Switzerland-Germany	7	335	203	1	[31]
Austria	6	315	223	-	[58]
Australia	4	361	45	-	[33]
Uruguay	4	69	-	-	[57]
Argentina	21	129	17	2	[17]
China	7	31	15	2	[80]

## References

[1] Forneck A., Huber L. (2009). (A)sexual reproduction—A review of life cycles of grape phylloxera, *Daktulosphaira vitifoliae*. Entomol. Exp. Appl..

[2] Powell K.S., Cooper P.D., Forneck A., Johnson S.N., Hiltpold I., Turlings T.C.J. (2013). The biology, physiology and host-plant interactions of grape phylloxera *Daktulosphaira vitifoliae*. Advances in Insect Physiology.

[3] Kellow A.V., Sedgley M., Van Heeswijck R. (2004). Interaction between *Vitis vinifera* and grape *phylloxera*: Changes in root tissue during nodosity formation. Ann. Bot..

[4] Forneck A., Kleinmann S., Blaich R., Anvari S.F. (2002). Histochemistry and anatomy of *phylloxera* (*Daktulosphaira vitifoliae*) nodosities on young roots of grapevine (*Vitis* spp.). Vitis.

[5] Nabity P.D., Haus M.J., Bernbaum M.R., De Lucia E.H. (2013). Leaf-galling *phylloxera* on grapes reprograms host metabolism and morphology. Proc. Natl. Acad. Sci. USA.

[6] Granett J., Walker M.A., Kocsis L., Omer A.D. (2001). Biology and management of grape phylloxera. Annu. Rev. Entomol..

[7] Eitle M.W., Loacker J., Meng-Reiterer J., Schuhmacher R., Griesser M., Forneck A. (2019). Polyphenolic profiling of roots (*Vitis* spp.) under grape phylloxera (*D. vitifoliae* Fitch) attack. Plant Physiol. Biochem..

[8] Griesser M., Lawo N.C., Crespo-Martinez S., Schoedl-Hummel K., Wieczorek K., Gorecka M., Liebner F., Zweckmair T., Pavese N.S., Kreil D. (2015). Phylloxera (*Daktulosphaira vitifoliae* Fitch) alters the carbohydrate metabolism in root galls to allowing the compatible interaction with grapevine (*Vitis* ssp.) roots. Plant Sci..

[9] Wapshere A.J., Helm K.F. (1987). Phylloxera and *Vitis*: An experimentally testable coevolutionary hypothesis. Am. J. Enol. Vitic..

[10] Viala P., Ravaz L. (1903). American Vines (Resistant Stock): Their Adaptation, Culture, Grafting and Propagation.

[11] Bournier A. (1976). Grape insects. Annu. Rev. Entomol..

[12] Downie D.A. (2002). Locating the sources of an invasive pest, grape phylloxera, using a mitochondrial DNA gene genealogy. Mol. Ecol..

[13] This P., Lacombe T., Thomas M.R. (2006). Historical origins and genetic diversity of wine grapes. Trends Genet..

[14] Ollat N., Peccoux A., Papura D., Esmenjaud D., Marguerit E., Tandonnet J.P., Bordenave L., Cookson S.J., Barrieu F., Rossdeutsch L., Geros H., Chaves M.M., Medrano H., Delrot S. (2016). Rootstocks as a component of adaptation to environment. Grapevine in a Changing Environment: A Molecular and Ecophysiological Perspective.

[15] Riaz S., Pap D., Uretsky J., Laucou V., Boursiquot J.M., Kocsis L., Walker M.A. (2019). Genetic diversity and parentage analysis of grape rootstocks. Theor. Appl. Genet..

[16] Migliaro D., De Lorenzis G., Di Lorenzo G.S., De Nardi B., Gardiman M., Failla O., Brancadoro L., Crespan M. (2019). Grapevine non-*vinifera* genetic diversity assessed by SSR markers as a starting-point for new rootstock breeding programs. Am. J. Enol. Vitic..

[17] Arancibia C., Riaz S., Aguero C., Ramirez-Corona B., Alonso R., Buscema F., Martinez L., Walker M.A. (2018). Grape phylloxera (*Daktulosphaira vitifoliae* Fitch) in Argentina: Ecological associations to diversity, population structure and reproductive mode. Aust. J. Grape Wine Res..

[18] Lund K.T., Riaz S., Walker M.A. (2017). Population structure, diversity and reproductive mode of the grape phylloxera (*Daktulosphaira vitifoliae*) across its native range. PLoS ONE.

[19] Riaz S., Lund K.T., Granett J., Walker M.A. (2017). Population diversity of grape phylloxera in California and evidence for sexual reproduction. Am. J. Enol. Vitic..

[20] Corrie A.M., Buchanan G.A., Van Heeswijck R. (1997). DNA typing of populations of phylloxera (*Daktulosphaira vitifoliae* (Fitch)) from Australian vineyards. Aust. J. Grape Wine Res..

[21] Downie D.A. (2005). Evidence for multiple origins of grape *phylloxera* (*Daktulosphaira vitifoliae* Fitch) (Hemiptera: *Phylloxeridae*) in South African vineyards. Afr. Entomol..

[22] Yvon M., Peros J.P. (2003). Variation in aggressiveness and genetic diversity of grape phylloxera in Southern France. J. Int. Sci. Vigne Vin..

[23] Eitle M.W., Forneck A. (2017). Comparison of bioassays to biotype grape phylloxera (*Daktulosphaira vitifoliae* Fitch) on *Vitis* ssp. Vitis.

[24] Downie D.A., Fisher J.R., Granett J. (2001). Grapes, galls, and geography: The distribution of nuclear and mithocondrial DNA variation across host-plant species and regions in a specialist herbivore. Evolution.

[25] Corrie A.M., van Heeswijck R., Hoffmann A.A. (2003). Evidence for host-associated clones of grape phylloxera *Daktulosphaira vitifoliae* (Hemiptera: Phylloxeridae) in Australia. Bull. Entomol. Res..

[26] Forneck A., Walker M.A., Blaich R. (2001). Ecological and genetic aspects of grape phylloxera *Daktulosphaira vitifoliae* (Hemiptera: Phylloxeridae) performance on rootstock hosts. Bull. Entomol. Res..

[27] Forneck A., Powell K.S., Walker M.A. (2016). Scientific opinion: Improving the definition of grape *Phylloxera* biotypes and standardizing biotype screening protocols. Am. J. Enol. Vitic..

[28] Saxena R.C., Barrion A.A. (1987). Biotypes of insect pests of agricultural crops. Int. J. Trop. Insect Sci..

[29] Sunnucks P., de Barro P.J., Lushai G., Maclean N., Hales D. (1997). Genetic structure of an aphid studied using microsatellites: Cyclic parthenogenesis, differentiated lineages and host specialization. Mol. Ecol..

[30] Vorweck S., Forneck A. (2006). Reproductive mode of grape phylloxera (*Daktulosphaira vitifoliae*, Homoptera: *Phylloxeridae*) in Europe: Molecular evidence for predominantly asexual populations and a lack of gene flow between them. Genome.

[31] Forneck A., Mammerler R., Tello J., Breuer M., Muller J., Fahrentrapp J. (2019). First European leaf-feeding grape phylloxera (*Daktulosphaira vitifoliae* Fitch) survey in Swiss and German commercial vineyards. Eur. J. Plant Pathol..

[32] Lushai G., De Barro P.J., Sherratt D.T.N., Maclean N. (1998). Genetic variation within a parthenogenetic lineage. Insect Mol. Biol..

[33] Corrie A.M., Crozier R.H., Heeswijck R.V., Hoffmann A.A. (2002). Clonal reproduction and population genetic structure of grape phylloxera, *Daktulosphaira vitifoliae*, in Australia. Heredity.

[34] Rollins L.A., Woolnough A.P., Sherwin W.B. (2006). Population genetic tools for pest management: A review. Wildl. Res..

[35] MacDonald C., Loxdale H. (2004). Molecular markers to study population structure and dynamics in beneficial insects (predators and parasitoids). Int. J. Pest Manag..

[36] Hoffmann M., Ruehl E.H., Eisenbeis G., Huber L. (2015). Indications for rootstock related ecological preferences of grape phylloxera (*Daktulosphaira vitifoliae* Fitch). Vitis.

[37] Turley M., Granett J., Omer A.D., De Benedictis J.A. (1996). Grape phylloxera (Homoptera: Phylloxeridae) temperature threshold for establishment of feeding sites and degree-day calculations. Environ. Entomol..

[38] Korosi G.A., Mee P.T., Powell K.S. (2012). Influence of temperature and humidity on mortality of grapevine phylloxera *Daktulosphaira vitifoliae* clonal lineages: A scientific validation of a disinfestation procedure for viticultural machinery. Aust. J. Grape Wine Res..

[39] Corrie A.M., Hoffmann A.A. (2004). Fine-scale genetic structure of grape phylloxera from the roots and leaves of *Vitis*. Heredity.

[40] Vorweck S., Forneck A. (2007). Analysis of genetic variation within clonal lineages of grape phylloxera (*Daktulosphaira vitifoliae* Fitch) using AFLP fingerprinting and DNA sequencing. Genome.

[41] Wilson A.C.C., Sunnucks P., Hales D.F. (2003). Heritable genetic variation and potential for adaptive evolution in asexual aphids (Aphidoidea). Biol. J. Linn. Soc..

[42] Lin H., Walker M.A., Hu R., Granett J. (2006). New simple sequence repeat loci for the study of grape phylloxera (Daktulosphaira vitifoliae) genetics and host adaptation. Am. J. Enol. Vitic..

[43] Benheim D., Rochfort S., Robertson E., Potter I.D., Powell K.S. (2012). Grape phylloxera (*Daktulosphaira vitifoliae*) —A review of potential detection and alternative management options. Ann. Appl. Biol..

[44] Zhu H., Sun Q., Du Y., Gao Z., Zhai H. (2015). Detection of grape phylloxera on grapevine roots with diagnostic polymerase chain reaction methods targeted to the internal transcribed space region 2 nuclear gene. Aust. J. Grape Wine Res..

[45] Giblot-Ducray D., Correll R., Collins C., Nankivell A., Downs A., Pearce I., McKay A.C., Ophel-Keller K.M. (2016). Detection of grape phylloxera (*Daktulosphaira vitifoliae* Fitch) by real-time quantitative PCR: Development of a soil sampling protocol. Aust. J. Grape Wine Res..

[46] Herbert K., Powell K., McKay A., Di Hartley H., Ophel-Keller K.M., Schiffer M., Hoffmann A. (2008). Developing and testing a diagnostic probe for grape phylloxera applicable to soil samples. J. Econ. Entomol..

[47] Triska M.D., Powell K.S., Collins C., Pearce I., Renton M. (2018). Accounting for spatially heterogeneous conditions in local-scale surveillance strategies: Case study of the biosecurity insect pest, grape phylloxera (*Daktulosphaira vitifoliae* (Fitch)). Pest Manag. Sci..

[48] Behura S.K. (2006). Molecular marker systems in insects: Current trends and future avenues. Mol. Ecol..

[49] Sunnucks P. (2000). Efficient genetic markers for population biology. Trends Ecol. Evol..

[50] Caterino M.S., Cho S., Sperling F.A.H. (2000). The current state of insect molecular systematics: A thriving Tower of Babel. Annu. Rev. Entomol..

[51] Loxdale H.D., Lushai G. (1998). Molecular markers in entomology. Bull. Entomol. Res..

[52] Lunt D.H., Zhang D.-X., Szymura J.M., Hewitt G.M. (2006). The insect cytochrome oxidase I gene: Evolutionary patterns and conserved primers for phylogenetic studies. Insect Mol. Biol..

[53] Lin H., Downie D.A., Walker M.A., Granett J., English-Loeb G. (1999). Genetic structure in native populations of grape phylloxera (Homoptera: Phylloxeridae). Ann. Entomol. Soc. Am..

[54] Fong G., Walker M.A., Granett J. (1995). RAPD assessment of California phylloxera diversity. Mol. Ecol..

[55] Forneck A., Walker M.A., Blaich R. (2000). Genetic structure of an introduced pest, grape phylloxera (*Daktulosphaira vitifoliae* Fitch), in Europe. Genome.

[56] Downie D.A. (2004). Phylogeography in a galling insect, grape *phylloxera*, *Daktulosphaira vitifoliae* (*Phylloxeridae*) in the fragmented habitat of the Southwest USA. J. Biogeogr..

[57] Bao L.V., Scatoni I.B., Gaggero C., Gutierrez L., Monza J., Walker M.A. (2015). Genetic diversity of grape phylloxera leaf-galling populations on *Vitis* species in Uruguay. Am. J. Enol. Vitic..

[58] Forneck A., Anhalt U.C.M., Mammerler R., Griesser M. (2015). No evidence of superclones in leaf-feeding forms of austrian grape phylloxera (*Daktulosphaira vitifoliae*). Eur. J. Plant. Pathol..

[59] Toth H.L., Taller J., Cernak I., Feher E., Kocsis L. (2007). Diversity of Hungarian grape phylloxera (*Daktulosphaira vitifoliae* Fitch) populations. Acta Hortic..

[60] Lynch M., Milligan B.G. (1994). Analysis of population genetic structure with RAPD markers. Mol. Ecol..

[61] Downie D.A. (2000). Patterns of genetic variation in native grape phylloxera on two sympatric host species. Mol. Ecol..

[62] Mueller U.G., Wolfenwarger L.L. (1999). AFLP genotyping and fingerprinting. Trends Ecol. Evol..

[63] Vos P., Hogers R., Bleeker M., Reijans M., van de Lee T., Hornes M., Frijters A., Pot J., Peleman J., Kuiper M. (1995). AFLP: A new technique for DNA fingerprinting. Nucl. Acids Res..

[64] Van de Zande L., Bijlsma R. (1995). Limitations of the RAPD technique in phylogeny reconstruction in *Drosophila*. J. Evol. Biol..

[65] Galtier N., Nabholz B., Glemin S., Hurst G.D.D. (2009). Mitochondrial DNA as a marker of molecular diversity: A reappraisal. Mol. Ecol..

[66] Hebert P.D.N., Ratnasingham S., De Waard J.R. (2003). Barcoding animal life: Cytochrome *c* oxidase subunit 1 divergences among closely related species. Proc. R. Soc. Lond. B Biol. Sci..

[67] Wang X.-Y., Yang X.-M., Lu B.R., Zhou L.-H., Wu K.-M. (2017). Genetic variation and phylogeographic structure of the cotton aphid, *Aphis gossypii*, based on mitochondrial DNA and microsatellite markers. Sci. Rep..

[68] Miranda E.A., Batalha-Filho H., Congrains C., Carvalho A.F., Ferreira K.M., del Lama M.A. (2016). Phylogeography of *Partamona rustica* (Hymenoptera, Apidae), an endemic stingless bee from the neotropical dry forest diagonal. PLoS ONE.

[69] Folmer O., Black M., Hoeh W., Lutz R., Vrijenhoek R. (1994). DNA primers for amplification of mitochondrial cytochrome *c* oxidase subunit I from diverse metazoan invertebrates. Mol. Mar. Biol. Biotechnol..

[70] Hebert P.D.N., Cywinska A., Ball S.L., deWaard J.R. (2003). Biological identifications through DNA barcodes. Proc. R Soc. Lond. B Biol. Sci..

[71] Cummings M.A., Krafsur E.S. (2005). Spatial diversity in mitochondrial cytochrome c oxidase in house flies. Med. Vet. Entomol..

[72] Davey J.W., Hohenlohe P.A., Etter P.D., Boone J.Q., Catchen J.M., Blaxter M.L. (2011). Genome-wide genetic marker discovery and genotyping using next-generation sequencing. Nat. Rev. Genet..

[73] Ellegren H. (2004). Microsatellites: Simple sequences with complex evolution. Nat. Rev..

[74] This P., Jung A., Boccacci P., Borrego J., Botta R., Costantini L., Crespan M., Dangl G.S., Eisenheld C., Ferreira-Monteiro F. (2004). Development of a standard set of microsatellite reference alleles for identification of grape cultivars. Theor. Appl. Genet..

[75] Schlötterer C. (2000). Evolutionary dynamics of microsatellite DNA. Chromosoma.

[76] Riaz S., Lund K., Lin H., Walker M.A. (2014). Development and characterization of a large set of microsatellite markers for grape phylloxera (*Daktulosphaira vitifoliae* Fitch). Vitis.

[77] Umina P.A., Corrie A.M., Herbert K.S., White V.L., Powell K.S., Hoffmann A.A. The Use of DNA Markers for Pest Management—Clonal Linages and Population Biology of Grape phylloxera. Proceedings of the III International Grapevine Phylloxera Symposium.

[78] Islam M.S., Roush T.L., Walker M.A., Granett J., Lin H. (2013). Reproductive mode and fine-scale population genetic structure of grape *phylloxera* (*Daktulosphaira vitifoliae*) in a viticultural area in California. BMC Genet..

[79] Forneck A., Dockner V., Mammerler R., Powell K.S., Kocsis L., Papura D., Fahrentrapp J., Riaz S., Walker M.A. (2017). PHYLLI—An international database for grape *phylloxera* (*Daktulosphaira vitifoliae* Fitch). IOBC-WPRS Bull..

[80] Sun Q.-H., Chen Y.-C., Du Y.-P., Zhai H. (2011). Genetic structure of grape phylloxera in China. Acta Hortic..

[81] Temnykh S., DeClerck G., Lukashova A., Lipovich L., Cartinhour S., McCouch S. (2001). Computational and experimental analysis of microsatellites in rice (*Oryza sativa* L.): Frequency, length variation, transposon associations, and genetic marker potential. Genome Res..

[82] Bonin A., Bellemain E., Bronken Eidesen P., Pompanon F., Brochmann C., Taberlet P. (2004). How to track and assess genotyping errors in population genetics studies. Mol. Ecol..

[83] Selkoe K.A., Toonen R.J. (2006). Microsatellites for ecologists: A practical guide to using and evaluating microsatellite markers. Ecol. Lett..

[84] Estoup A., Jarne P., Cornuet J.-M. (2002). Homoplasy and mutation model at microsatellite loci and their consequences for population genetics analysis. Mol. Ecol..

[85] Ekblom R., Galindo J. (2011). Applications of next generation sequencing in molecular ecology of non-model organisms. Heredity.

[86] Schwarz D., Robertson H.M., Feder J.L., Varala K., Hudson M.E., Ragland G.J., Hahn D.A., Berlocher S.H. (2009). Sympatric ecological speciation meets pyrosequencing: Sampling the transcriptome of the apple maggot *Rhagoletis pomonella*. BMC Genom..

[87] Perry K.D., Pederson S.M., Baxter S.W. (2017). Genome-wide SNP discovery in field and laboratory colonies of Australian *Plutella* species. Bioarxiv.

[88] Silva-Brandao K.L., Neto e Silva O.A.B., Brandao M.M., Omoto C., Sperling F.A.H. (2015). Genotyping-by-sequencing approach indicates geographic distance as the main factor affecting genetic structure and gene flow in Brazilian populations of *Grapholita molesta* (Lepidoptera, Tortricidae). Evol. Appl..

[89] Black W.C., Vontas J.G. (2007). Affordable assays for genotyping single nucleotide polymorphisms in insects. Insect Mol. Biol..

[90] Delmotte F., Papura D., Rispe C., Legeai F., Jaquiery J., Bretaudeau A., Tagu D., Powell K.S., Forneck A. (2014). The grape phylloxera genome sequencing project. Acta Hortic..

[91] Stucki D., Malla B., Hostettler S., Huna T., Feldmann J., Yeboah-Manu D., Borrell S., Fenner L., Comas I., Coscolla M. (2012). Two new rapid SNP-typing methods for classifying *Mycobacterium tuberculosis* complex into the main phylogenetic lineages. PLoS ONE.

[92] Morin P.A., Martien K.K., Taylor B.L. (2009). Assessing statistical power of SNPs for population structure and conservation studies. Mol. Ecol. Resour..

[93] Van Inghelandt D., Melchinger A., Lebreton C., Stich B. (2010). Population structure and genetic diversity in a commercial maize breeding program assessed with SSR and SNP markers. Theor. Appl. Genet..

[94] Tello J., Roux C., Chouiki H., Laucou V., Sarah G., Weber A., Santoni S., Flutre T., Pons T., This P. (2019). A novel high-density grapevine (*Vitis vinifera* L.) integrated linkage map using GBS in a half-diallel population. Theor. Appl. Genet..

[95] Elshire R.J., Glaubitz J.C., Sun Q., Poland J.A., Kawamoto K., Buckler E.S., Mitchell S.E. (2011). A robust, simple genotyping-by-sequencing (GBS) approach for high diversity species. PLoS ONE.

[96] Anderson C.J., Tay W.T., McGaughran A., Gordon K., Walsh T.K. (2016). Population structure and gene flow in the global pest, *Helicoverpa armigera*. Mol. Ecol..

[97] Schmidt J.M., Battlay P., Gledhill-Smith R.S., Good R.T., Lumb C., Fournier-Level A., Robin C. (2017). Insights into DDT resistance from the *Drosophila melanogaster* genetic reference panel. Genetics.

[98] Magwire M.M., Fabian D.K., Schweyen H., Cao C., Longdon B., Bayer F., Jiggins F.M. (2012). Genome-wide association studies reveal a simple genetic basis of resistance to naturally coevolving viruses in *Drosophila melanogaster*. PLoS Genet..

[99] Rispe C., Legeai F., Papura D., Bretaudeau A., Hudaverdian S., Le Trionnaire G., Tagu D., Jaquiery J., Delmotte F. (2016). De novo transcriptome assembly of the grapevine phylloxera allows identification of genes differentially expressed between leaf- and root-feeding forms. BMC Genom..

[100] Delmotte F., Forneck A., Powell K., Rispe C., Tagu D. Proposal to sequence the genome of the grape Phylloxera (*Daktulosphaira vitifoliae* Fitch). http://bipaa.genouest.org/is/wp-content/uploads/2015/10/White-Paper-Phylloxera_25may2011-1.pdf.

[101] Forneck A., Jin Y., Walker A., Blaich R. (1999). Karyotype studies on grape phylloxera (*Daktulosphaira vitifoliae* Fitch). Vitis.

